# The Obstetric Bag

**Published:** 1862-09

**Authors:** Robert Barnes

**Affiliations:** Fellow of the Royal College of Physicians, Physician to the Royal Maternity Charity, Lecturer on Midwifery at St. Thomas’s Hospital


### THE OBSTETRIC BAG.

### A DESCRIPTION OF THE INSTRUMENTS USED IN OPERATIVE MIDWIFERY.

*A Lecture delivered in St. Thomas's Hospital,*

By ROBERT BARNES, M.D.,
Fellow of the Royal College of Physicians, Physician to the Royal Maternity Charity, Lecturer on Midwifery at St. Thomas's Hospital.

---

Gentlemen:—Before demonstrating the operations which you may be called upon to perform in the practice of midwifery, it will be useful to describe the instruments with which those operations are performed. In no department of surgery is there a greater variety, or a more embarrassing confusion of instruments, than in midwifery. I imagine it is this very confusion, as much as the ambition of appearing original, that has driven many practitioners to devise fresh instruments for their own use. Thus has confusion been worse confounded; and not seldom have men unwitting committed the blunder of inventing over again an instrument which had long been known to others.

The International Exhibition will give you some faint idea of the extent of the modern obstetric armamentarium. An inspection of the surgical instruments sent by the principal makers of Europe may be recommended to every student and practitioner. The observer will be struck, perhaps, at first with the multiplicity of the means contrived for the accomplishment of the same end. But a more analytical study will suggest to him the further reflection that in this multiplicity of forms of instruments bearing the same names there may lie a deeper purpose and a more instructive lesson. The greater number of the modifications made in the forceps, for example, reveal successive attempts to overcome some difficulty in practice, or even to extend the applications and increase the usefulness of this admirable instrument. You will, again, be led by the examination of the instruments, which are the visible expression of the thoughts of their designers, to inquire into the peculiarities and doctrines of different practioners and of various schools. It is curious to observe how closely certain primary types of instruments have been preserved in different countries. The straight forceps, with the short handles of Chamberlen, reappears it its essential characters in the instrument of Chapman, Smellie, Denman, and other English teachers; and even cherished by some at the present day. In France, the latest modifications of Dubois and Pajot still bear a striking resemblance to the long forceps with double curve and long handles of Levret. The forceps by birthright is an English instrument. But it has not been simply naturalized in France. Our continental brethren have impressed upon it a new idea, and made it capable of accomplishing much more than the old English model could do. Then, again, you will see the instruments in the foreign cases which are altogether unknown to English practice—instruments which are not to be procured from our best cutlers at home. There is for example, the cephalotribe, for crushing down the head to dimensions that will admit of dragging through a greatly contracted pelvis. You may think it singular that this powerful and formidable weapon should be needed abroad, and that it should not have been found necessary here. You will turn to the instruments used in England to overcome those difficulties which, on the continent generally, are encountered by other means. This comparative study will, in this and many other ways, prove exceedingly interesting and instructive. I cannot enter into it fully now, as my immediate duty is to explain to you the construction and uses of the several pieces of such a selection of instruments as I have found to be best adapted for the exigences of practice.

And first of all I would say a few words about that most essential and most universally applicable obstetric instrument, the human hand. In ordinary labor of course it is the only instrument required; but it is also the sole instrument called for in many of the greatest difficulties that occur in obstetric practice. In malpresentations—in many cases of contracted or deformed pelvis—in not a few cases where, after craniotomy, the crotchet and craniotomy forceps have failed to deliver—the bare hand affords a safe and ready extrication.

I believe that if manual dexterity were more cultivated than it is, lives that are now lost would be saved, and certainly many tedious and dangerous instrumental operations would be avoided. It would be both instructive and curious to carry our minds back to the days when the forceps and other obstetric instruments were unknown, and to confront the problem which our predecessors—such men as Ambroise, Pare, Guillemeau, and others—had to solve: namely, how to deliver a woman with a deformed pelvis without instruments. That they did accomplish in many instances with the unarmed hand what we now do by the aid of various weapons there can be no doubt. If this implies greater poverty of resources on their part, it not the less implies also greater manual skill. I am confident that the possession of instruments, especially of the craniotomy instruments, has led within the last century, to a neglect of the proper uses of the hands, which is much to be deplored. Indeed, it may be said that the more certain instruments are perfected, the more they will be trusted and resorted to, until we are in danger of greatly exaggerating their value. The question is not merely one of choice between two methods of obtaining the same end; it is frequently one between life and death. The perforator and crotchet for example, kill the child, where the hand would sometimes save it, and that without adding to the mother's risk. I say it deliberately, after no mean experience in obstetric operations, that what is most wanted at the present day is not the improvement or invention of instruments, but a more careful and scientific cultivation of the powers of the hand, and a judicious selection of the best models of existing instruments.

Obstetric surgery has this peculiarity: its operations are carried on in the dark, our only guide being the information conveyed by the sense of touch. The mind's eye travels on the fingers' ends. The hand thus possesses an inestimable superiority over all over instruments. Its every movement is regulated by consciousness. If used with ordinary discretion and skill, it can hardly inflict injury.

After expending a world of ingenuity upon the invention and perfecting of obstetric instruments, it is time to ascend a little the stream of knowledge, and to endeavor to recover from the teaching of our forefathers their secret of *chirurgery;* to regain, if not to extend, that power over the great instrument which is the surgeon's most trusty aid, and from which he derives his name.

In providing yourselves with a set of instruments, I would recommend you to discard the old rolling up leathern case, or *trousseau.* It is an inconvenient contrivance. It only carries a restricted number of things. I find a modification of the travelling leather bag far more useful. It will carry not only the selected instruments ordinarily required, but you can always put anything else in it that may be specially indicated. It will hold bottles; serve to bring away pathological specimens. In short, it makes a perfect accoucheur's vade-mecum. And if you turn out the obstetric furniture, you have a traveling bag again. The following is a list of the ordinary furniture of the Obstetric Bag:—

1. A pair of long double-curved forceps.
2. A craniotome, or perforator.
3. A crotchet.
4. A craniotomy forceps.
5. A blunt-ended straight bistoury, with a cutting edge of three-quarters of an inch, to incise the os uteri in cases of extreme contraction or cicatrization.
6. A Higginson's syringe, fitted on my plan, with a long uterine tube, which serves for the injection of iced water, etc., to arrest hæmorrhage, and also serves to expand.
7. A set of my caoutchouc uterine dilators, for the induction of premature labor, and the acceleration of labor, as in cases of placenta prævia, convulsions, etc.
8. A flexable male catheter. (The short silver female catheter is often useless, and is generally less convenient than the flexable male catheter.)
9. A pair of scissors.
10. Thread.
11. Some yards of tape.

*Medicines.*

1. Chloroform and inhaler. 3. Hofman's anodyne.
2. Laudanum. 4. Ergot of Rye.
5. A solution of perchloride of iron.

The medicines can be fitted in small pockets at the upper part of the bag.

These are the things always kept in my bag ready for use. If you should want anything else, the bag will hold it.

I will now say a few words in explanation of the construction and uses of some of the instruments enumerated:

*The Forceps.*—Next to a skilful hand, the possession of a good forceps is of the greatest importance to the practitioner of midwifery. A good forceps is in many cases a substitute for the perforator; and in many others it may save the mother hours of torture, and the dangers that attend upon protracted labor. Those who restrict themselves to the use of the short forceps, or even to a long forceps with the single or cranial curve, must inevitably expose many patients to the dangers of flooding, of convulsions, of protracted labor, of jamming the lower segment of the uterus between the child's head and the sharp pelvic brim; or, to obviatd these perils to the mother, they must sacrifice her child. Every one will grant that craniotomy is the confession of the impotency of our art to save. It ought to be regarded as the last resource. Every effort should be made to improve these instruments and operations which obviate the necessity of resorting to this cruel alternative of destroying the child. The success of these saving operations often, indeed, depends upon good instruments being skilfully employed. Without indulging in the flattering hope that craniotomy will ever be exploded, still I do think that the degree to which this brutal operation shall be minimized must be accepted as a measure of the improvement of obstetric surgery. The great agent of this reduction must be the forceps. And to answer the indications fully, this instrument must be of sufficient length and properly curved to reach the head arrested on the brim of the pelvis, and of sufficient power to arrest some compressive action upon the fœtal head to bring it through a moderately contracted space. If you will look at the French forceps, you will understand how it is that in France craniotomy is so rare, and why they pass at once from the forceps to the cephalotribe—an instrument so formidable that it seems only adapted for the most extreme cases of disproportion. Do they never witness abroad those minor degrees of disproportion in which, here, craniotomy is resorted to? Certainly such cases must and do occur. The inference we must draw is, that the forceps is made to do the work of the perforator; that by means of this instrument our brethren in France are enabled to rescue from the perforator a large class of intermediate cases ranging between the normal proportions and the extreme disproportions which call for the cephalotribe or the Cæsarean section.

The French forceps, with its powerful blades and long handles, present a formidable appearance to eyes accustomed to the small, single-handed forceps of this country. If, however, I should ever be tempted to lay aside an instrument which has served me well on many occasions, it will probably be to take up the improved forceps of Dubois or that of Pajot. The great bugbear of English operative midwifery is the absurd dread of possessing powerful instruments. Thus the blades of the forceps are made straight and short; the lock is placed close to the bow of the blades, and the handles are so short that they can only be grasped with one hand—all to carry out the idea that to make a safe instrument, it must be weak or powerless. There is no greater error than this. In the first place, a weak instrument is, by the mere fact of its weakness, restricted to a very limited class of cases. In the second place, if the instrument is weak, it calls for more muscular force on the part of the operator. Now, it is sometimes necessary to keep up a considerable degree of force for some time, and not seldom in a constrained position. Fatigue follows; the operator's muscles become unsteady; the hand loses its nicety of diagnostic touch, and that exactly balanced control over its movements which it is so important to preserve. Under these circumstances, he soon comes to the conclusion that he has used all the force that is justifiable; that the case is not fitted for the forceps, and takes up the horrid perforator; or he runs the risk of doing that mischief to avoid which his forceps was made weak. It is a transparent fallacy to conclude that an instrument is dangerous because it is powerful. If you have an excess of power, your need not use it; certainly you need not abuse it. Would it not be ridiculous to bring down the power of a man to the puny, unsteady strength of a boy, when there is work requiring power and skill to be done? Having force to spare, it is more easy to regulate the amount of force you employ; you gain more perfect command over your instrument; you need never exhaust your own muscular power. The golden rule in midwifery, as in all other affairs, is never to expend unnecessary force. The steam-hammer will strike with a force of many tons when its duty is to weld bars of iron into one mass; but when called upon to crack a nut, it will just break the shell without bruising the kernel. So in obstetric operations you will so exactly graduate the force at your disposal as to effect your purpose and nothing more. This faculty of accurate graduation depends, I repeat, upon having a reserve of force. Violence is the fault of struggling feebleness, not of conscious power. Moderation must emanate from the will of the operator; it must not be looked for from the imperfection of his instruments. One of the most valuable features of the French forceps is the length of the handles, which admits of grasping by both hands at once. It does not follow that, because of this facility, you are to throw the whole strength of both arms into the work of compression and extraction. The true use of a two-handled instrument is to enable one hand to assist, to relieve, to steady the other. By alternate action, the hands get rest, the muscles preserve their tone, and that accurate sense of resistance which is necessary to guide the surgeon in measuring the degree of force that is called for, or to warn him when to desist. The instrument I now show you, which I have used exclusively for several years, although less powerful than the French forceps, answers similar indications. It is, as I believe, as regards the form of the blades, the forceps of Mr. Roberton, of Manchester; but instrument-makers tell me that my instrument is different in other respects. The blades are double-curved, but both the pelvic and the cranial curves are slight; the length of the arc of the cranial bow is seven inches, thus adapting it to that elongation of the fœtal cranium which is commonly the result of slight pelvic contraction and protracted labor; then there is a straight shank an inch and a-half long, and a semicircular bow, the arc or diameter of which is also an inch and a-half long. This half circle in each blade, when opposed to its fellow, makes a ring. This ring is immediately in front of the lock. The lock is the commencement of the handles, which are four inches and a-quarter long. The handles might be lengthened with advantage. The straight shank and the ring make together a length of three inches between the springing of the bow and the lock. This effectually clears the lock from all risk of entanglement with the labia or perineum. The ring offers a most convenient hold for the forefinger, whilst the handle can be grasped either with the remainder of the same hand, or with the other hand. You have thus essentially a two-handed instrument, which can be worked with great nicety and economy of muscular force. This forceps is equally adapted for arrest at the brim, in the cavity, or near the outlet of the pelvis. It is quite superfluous to encumber yourselves with more than one instrument. If you consider that this forceps is made to seize a head above the brim, and to bring it down through the entire pelvic canal, you will see that it is of necessity fitted for application at any lower point of the pelvis. There are two more practical advantages possessed by this instrument: it adds nothing whatever to the risk of laceration to the perineum—a risk which is very serious with the straight forceps; owing to the pelvic curve, the upper or right blade can be introduced without dragging the patient's nates out of bed, or over the edge. The application of the instrument, indeed, requires very little disturbance of the patient.

You thus avoid inflicting that terror which attends troublesome preparations. I have saved many children with this forceps, who had been otherwise doomed to perish by craniotomy. I have never seen injury inflicted by it upon the mother, as I have seen by the short forceps even in skilful hands. I entertain a confident opinion that the short straight forceps is destined to disappear from obstetric practice..

*The Craniotomy Instruments.*—Many perforators and craniotomy-forceps employed are very defective. The perforators will not perforate, and the forceps will not hold. The point of the perforator should be straight. To curve the point is to throw the force out of its proper line. The shank should be long, not less than seven inches, to enable the point to reach above the brim of the pelvis, whilst the handles are clear of the external parts. The shanks should diverge well, so as to admit the entire hand between the handles. Here, again, you will find the advantage of power in the instrument. It is the surest safeguard against slipping, against failure, against injuring the mother. This form of instrument was recommended to me by Dr. Oldham. It answers its purpose thoroughly. The best crotchet is that used in the Dublin Lying-in Hospital. I think that extraction by the craniotomy-forceps is generally preferable; but then you must have a good forceps. I can understand that having to choose between a good crotchet and a bad craniotomy-forceps the preference should be given to the former. The craniotomy-forceps I show you is an excellent and powerful instrument. It is a modification made by Messrs. Weiss, under my direction of the instruments of Professors Simpson and Murphy. The blades can either be introduced singly or as one instrument; but, in truth, this forceps is very rarely called for. In the first place, craniotomy ought to be rare; and, in the second place, I have often found it far more easy and expeditious to deliver a child after perforation by turning than by the crotchet or forceps.

What I have said to-day is for the express purpose of assisting in the selection of a good set of obstetric instruments. I am sure I have more frequently seen practitioners fail in operations they have undertaken from want of good models of instruments than from want of skill. Hence the importance I attach to this point. The mode of using the instrument I shall demonstrate hereafter.—*London Lancet.*

